# Identification of gene specific cis-regulatory elements during differentiation of mouse embryonic stem cells: An integrative approach using high-throughput datasets

**DOI:** 10.1371/journal.pcbi.1007337

**Published:** 2019-11-04

**Authors:** M. S. Vijayabaskar, Debbie K. Goode, Nadine Obier, Monika Lichtinger, Amber M. L. Emmett, Fatin N. Zainul Abidin, Nisar Shar, Rebecca Hannah, Salam A. Assi, Michael Lie-A-Ling, Berthold Gottgens, Georges Lacaud, Valerie Kouskoff, Constanze Bonifer, David R. Westhead

**Affiliations:** 1 School of Molecular and Cellular Biology, Faculty of Biological Sciences, University of Leeds, Leeds, United Kingdom; 2 Wellcome Trust & MRC Cambridge Stem Cell Institute and Cambridge Institute for Medical Research, University of Cambridge, Cambridge, United Kingdom; 3 Institute for Cancer and Genomic Sciences, College of Medical and Dental Sciences, University of Birmingham. Birmingham, United Kingdom; 4 CRUK Manchester Institute, University of Manchester, Manchester, United Kingdom; 5 Division of Developmental Biology and Medicine, The University of Manchester, Manchester, United Kingdom; University of Wisconsin, Madison, UNITED STATES

## Abstract

Gene expression governs cell fate, and is regulated via a complex interplay of transcription factors and molecules that change chromatin structure. Advances in sequencing-based assays have enabled investigation of these processes genome-wide, leading to large datasets that combine information on the dynamics of gene expression, transcription factor binding and chromatin structure as cells differentiate. While numerous studies focus on the effects of these features on broader gene regulation, less work has been done on the mechanisms of gene-specific transcriptional control. In this study, we have focussed on the latter by integrating gene expression data for the *in vitro* differentiation of murine ES cells to macrophages and cardiomyocytes, with dynamic data on chromatin structure, epigenetics and transcription factor binding. Combining a novel strategy to identify communities of related control elements with a penalized regression approach, we developed individual models to identify the potential control elements predictive of the expression of each gene. Our models were compared to an existing method and evaluated using the existing literature and new experimental data from embryonic stem cell differentiation reporter assays. Our method is able to identify transcriptional control elements in a gene specific manner that reflect known regulatory relationships and to generate useful hypotheses for further testing.

## Author summary

The inherited information in our DNA genomes is a code which defines both the functional units (proteins, nucleic acids etc.), and patterns of their usage, necessary to make life. The genome in mammals, such as man and mouse, has genes which code for about 20000 different proteins, but the usage of these proteins differs in each different type of cell within these complex multicellular organisms. How this differential usage is controlled in known as genetic regulation, and that is what we study here. We know that the details lie in how genes are turned on and off, but until the advent of high-throughput sequencing technology a genome-wide study was nearly impossible. Further complicating our efforts to understand genetic regulation is the involvement of parts of the genome that were previously deemed junk. In this work, we have focussed on how the genes are controlled at various developmental stages in mouse, by looking at the sequencing data from different regulatory mechanisms such as protein binding and local changes to DNA packaging etc. On a gene-by-gene basis, we have built statistical models that predict how genes are controlled when cells develop. These predictions provide a focus for future experimental studies of genetic regulation.

## Introduction

The fate of a cell is determined by dynamics in the expression of genes, a process that is regulated at the highest level by the control of transcription [1, 2]. Cell differentiation at the genome level involves a complex interplay of processes [3], including DNA binding by transcription factors (TFs) [4] and changes in the structure of chromatin and DNA, for example, DNA methylation and epigenetic modifications of amino acids of the histones [5]. With the recent developments in high throughput sequencing (HTS) researchers have been able to study the genome-wide implications of these processes in various cell types and organisms [6]. From these studies, we have gained global insights into transcriptional regulation, such as the relationship between chromatin accessibility around the promoter region and gene expression [7, 8], the prevalence of histone modifications such as H3K27ac and H3K9ac near expressed genes [9], the presence of H3K27me3 modification near transcriptionally repressed genes [10] and the binding of master regulators to genes that are often associated with lineage differentiation [11, 12].

While patterns in regulatory mechanisms have been identified, much of the detail of the regulatory system still remains unknown, owing to the complex structure of the transcription machinery. Of note is the implication of non-coding regions of the genome of higher eukaryotes in transcriptional and post-transcriptional regulation of protein coding genes [4, 13]. Equally, it is becoming increasingly clear that much of the dynamics in gene expression is governed not by the regulatory input at promoters but to distal sites including enhancers [14, 15]. Enhancers have been a subject of interest in recent years because of their major role in transcriptional control of tissue specific gene expression programmes [14, 16, 17]. There are several proposed models for the mechanism of enhancer interaction with the RNA Polymerase II (RNAP) machinery over large distances to control gene expression [18], whereby the looping model is becoming widely accepted [19]. All the models propose binding of TFs to both enhancer and promoter regions and involve formation of multi-protein complexes. This aspect of combinatorial binding of TFs to control regions is well established [12, 20, 21].

Enhancer regions may be identified experimentally by reductionist approaches such as mutations in genomic regions associated with loss of expression of a nearby gene, and by using reporter assays to investigate transcriptional enhancement in cell lines or *in vivo*. There has also been significant interest in the identification of enhancers using theoretical methods. These can be based on characteristics of the DNA sequence, particularly conservation, and the large-scale availability of ChIP-seq data for well-known enhancer characteristics (the H3K27ac and H3K4me1 chromatin marks, binding of the EP300 coactivator protein etc.) has led to more sophisticated methods using machine learning to combine information from different sources. These efforts have been recently reviewed [2, 22–24], and it has been shown that binding by transcription factors is a highly specific indicator of enhancer activity.

Extending the problem of identification is the issue of mapping those enhancer elements to the genes they regulate. It is now widely accepted that physical enhancer-promoter (EP) interactions are required for transcriptional control [25, 26]. A range of related experimental techniques (3C, 4C, HiC, ChIA-PET, capture C) classed as chromosome conformational capture assays enable us to determine the three-dimensional conformations of specified regions or the whole of the genome [26] and EP interactions are likely to be a subset of the numerous chromosomal interactions identified [4, 27]. These techniques are being rapidly developed and the increasing availability of data sets, particularly in human cells, is leading to interesting insights [28, 29].

The enhancer mapping problem has also received theoretical attention, and extending earlier work [30–35] based on epigenetic activity correlations between enhancers and their putative target genes, more recent studies have taken advantage of the increasing availability of experimental data for three-dimensional genome interactions [36, 37]. For human cells, projects like ENCODE [38] have made available genome scale data on chromatin structure, transcription factor binding and gene expression, enabling the development of methods to predict EP interactions from them, with training and validation employing appropriate 3D interaction data [30–32, 39]. For example, RIPPLE uses a Random Forest classifier with 5C data and a selected set of highly predictive features for identification of enhancers, while JEME [40] considers the joint effects of multiple predicted enhancers on gene expression and earlier similar methods based predictions on different features [37, 41, 42]. Recently, Xi and Beer [43] have raised concerns about the validation and estimated accuracy of machine learning methods that rely on 3D interaction data to predict enhancers. They focussed on Target Finder [42] which uses Hi-C data, and they advocate careful consideration of cross-validation methods since the coarser resolution of this data can lead to groups of potential enhancers all exhibiting the same pattern of promoter interactions. Alternative approaches have employed the correlation of enhancer activity and bidirectional enhancer transcription [44], and in other model organisms such as Drosophila, the emphasis has been on sequence features of the predicted enhancers linking to developmental gene expression patterns [45, 46].

In this study, we address the related problem of identifying those gene specific cis control elements, many of which are expected to be enhancers, which are most relevant in controlling the expression of key genes involved in cellular lineage specification and progressive lineage restriction. Our focus is a data set from mouse, where three-dimensional interaction data are not available in most of the cell types. This method integrates chromatin immunoprecipitation, DNaseI-seq and RNA-seq data, and analyses two branching pathways of *in vitro* cellular differentiation, leading from embryonic stem (ES) cells to the myeloid blood lineage (macrophages) in one branch and to cardiac cells (cardiomyocytes) in the other [15, 47]. Our method is based on correlating a measure of the activity of a candidate cis-regulatory element (CRE), as indicated by transcription factor occupancy and chromatin structure/modification, with the pattern of gene expression. We introduce a new concept of cis-regulatory element communities (coCREs), which are genomic regions that show correlated patterns of activity and transcription factor binding and are considered together for robust model building. We compare the observations with known and predicted sets of enhancers and furthermore with the results from JEME trained on 3D interaction data from mouse embryonic stem cells.

Our study indicates a gene-specific coordinated binding of multiple master regulators during differentiation to control lineage specific expression of important genes, implying that the gene-centric approach may shed further light on the relationship between cell fate decisions and the underlying transcriptional landscape. We were able to successfully recapitulate regulation of cell type specific genes using previously known cis elements and also have been able to propose gene specific control mechanisms using novel regulatory regions identified through gene-specific expression modelling. Furthermore, we found that the genomic loci that are most predictive were characterised by high phylogenetic conservation, epigenomic activities and transcription factor binding. Finally, using reporter assays we were able to confirm two novel cis regulatory elements for genes *Nfe2* and *Sptbn1*. This statistical approach that works with fewer assays and does not require topological data, is simpler to apply, adapt and understand than machine learning approaches. Furthermore, by employing *in vitro* cell types and focussing on mouse, an important model organism for which relatively less information on enhancers is available, the data provided here is a valuable resource to the research community.

## Results and discussion

In order to study the mechanism of transcriptional control of genes involved in lineage specific cell fate decisions, we integrated gene expression and regulatory data from the two *in vitro* mouse differentiation pathways described above. Details of the associated cell types and data sets employed are given in Table 1. The results of integrating normalised expression data for protein coding transcripts are shown in (S1A–S1C Fig): both clustering and principal components analysis indicate effective integration where related cell types from both series (ES cells, ESC and CESC; mesoderm cells, MES and CMES) show clear similarity, and terminally differentiated macrophages emerge as the most different cell type, nevertheless still showing clear lineage development from the intermediate haemopoietic cells. From this data, we identified 9854 differentially expressed genes (for details see Methods), and their patterns of expression were clustered using *k*-means to produce 391 clusters at the optimal BIC (Bayesian Information Criterion) score (S1D Fig). For the purpose of predictive modelling we considered 3 sets of genes: the 17 TFs for which we have ChIP-seq data (the ‘TF set’), the 437 genes that co-cluster with the TF set (‘TF cluster set’) and finally the remaining differentially expressed genes (‘DE gene set’). Details of the gene sets and clustering are provided in S1 Table and S2 Fig.

**Table 1:** Datasets and cell types used in this study.

Pathway	Cell type	Description	ChIP-seq (H3K27Ac)	DNase1-seq	RNA-seq	ChIP-seq (TF)
**mESC → Macrophages**	ESC	ES cells	✓	✓	✓	NANOG, OCT4, SOX2, ESRRB
	MES	Mesoderm	✓	✓	✓	C/EBPB, ELK4, OCT4
	HB	Hemangioblast	✓	✓	✓	C/EBPB, GATA2, LMO2, TAL1
	HE	Hemogenic Endothelium	✓	✓	✓	C/EBPB, LMO2, TAL1, FLI1, MEIS1
	HP	Hematopoietic Progenitors	✓	✓	✓	C/EBPB, FLI1, GATA1, GATA2, GFI1, GFI1B, LMO2, PU.1, RUNX1, TAL1
	MAC	Macrophages	✓	✓	✓	C/EBPB, FLI1, LMO2, PU.1, RUNX1, TAL1
**mESC→ Cardiomyocytes**	CESC	ES cells	✓	✖	✓	✖
	CMES	Mesoderm	✓	✖	✓	✖
	CP	Cardiac Progenitors	✓	✖	✓	✖
	CM	Cardiomyocytes	✓	✖	✓	✖

**Fig 1:**
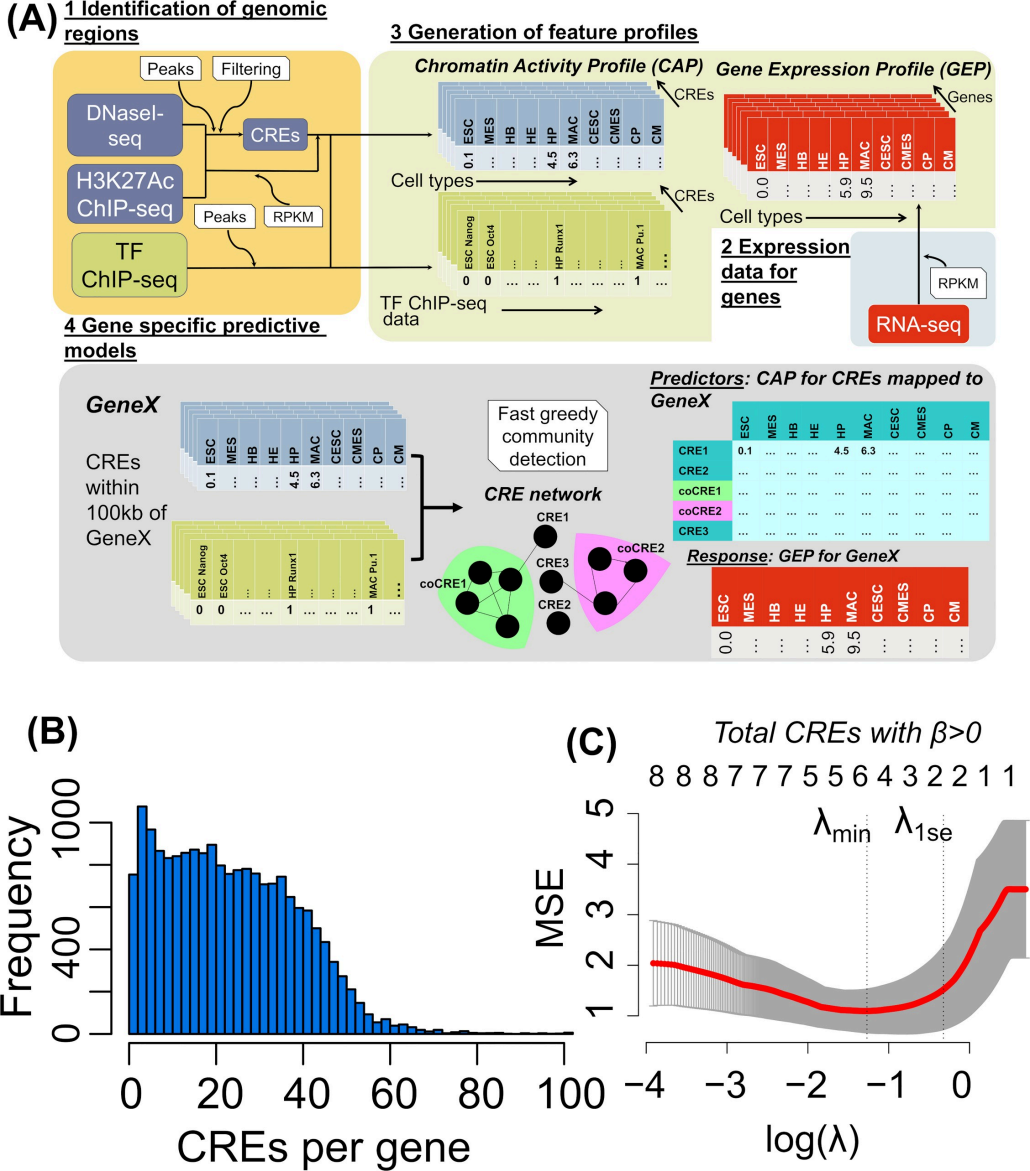
Gene-specific predictive models. (A) Schematic representation of the methodology involved in developing gene specific predictive models. 1. Integration of DNaseI-seq and H3K27ac to quantify the chromatin activity profile (CAP) in candidate cis regulatory elements (CREs). TF ChIP-seq data is used to generate the transcription factor binding profile (TFBP) to quantify the community effect of candidate CREs mapped to a specific gene. 2. Gene wise expression values are obtained as RPKMs to form gene expression profiles (GEPs). 3. CAPs, TFBPs and GEPs are generated for all the regions and genes in the analysis. 4. CAP and TFBP are integrated in order to generate gene specific CRE networks. A greedy community detection is performed in order to identify the communities of CREs (coCREs) in the networks. A new set of CAPs involving aggregate CAPs of the coCREs along with the individual CAPs for singleton CREs are used to predict the GEP for a specific gene. (B) Histogram showing the distribution of candidate CREs per gene within 100kB of the transcription start site over all genes in the study. (C) The plot shows the change in cross-validated Mean Squared Error (MSE) as a function of increasing *λ* for a predictive model of *Runx1* gene expression. The two vertical dotted lines show the two cut offs λ_*min*_ and λ_1*se*_. The total number of CREs with non-zero coefficients (*β*) at a given *λ* is shown above the plot.

The essence of the method presented here is to identify for each gene studied, a small set of CREs that best explain its pattern of expression (the ‘Gene Expression Profile’ (GEP)) from a relatively large set of candidate CREs. The methodology is illustrated in Fig 1A and full details can be found below and in Methods. In summary, for each gene, initial candidate CREs are genomic regions within 100 kBases (kB) of the transcription start site (TSS) with significant enrichment in H3K27ac ChIP-seq (a mark of active enhancers) data and/or a DNaseI-seq hypersensitive site (DHS). Candidate CREs are characterised by a chromatin activity profile (CAP, see Methods) across the cell types, derived from H3K27ac and DNase1-seq data, and also a TF binding profile (TFBP). This information is integrated into a CRE network, from which community CREs (coCREs) are identified (see Methods). The CAP of a coCRE is the average of its constituent CREs. A gene-specific penalised regression is used to choose CREs that best predict the gene’s expression profile (GEP) from the set of singleton and community CREs.

### Active chromatin regions as candidate CREs

The 262770 chromatin regions that are active (either H3K27ac enriched or a DNaseI Hypersensitive Site (DHS), see Methods sections), form our candidate CREs and are characterised in S3 Fig. A ‘chromatin event’ in a CRE is a H3K27ac peak or DHS peak in a particular cell type, and S3A Fig is a frequency plot for chromatin events in all candidate CREs. Many CREs have only one chromatin event over all cell types: in order to use only high confidence CREs we retained only those with more than one chromatin event (118688 regions given in S2 Table), and with enrichment level (either H3K27ac or DNaseI-seq) in at least one cell type higher than the lower quartile in the distribution of enrichment levels (S3D and S3E Fig, 82165 regions). S3B Fig shows that the sizes of the CREs used in this study are around 1–2 kB in width and S3C Fig shows the distribution of their conservation scores. It can be seen that majority of CREs have low conservation across the vertebrates, while a selective subset of regions is highly conserved. From this initial set of CREs, coCREs are identified as given below.

### Community CREs (coCREs)

Fig 1B shows that genes may have many candidate CREs, ranging from zero to 100 with an average of around 18. This exemplifies the challenge of gene specific models that the number of potential predictors of gene expression exceeds the number of measurements, and calls for specific methods such as penalized regression (see below). However, preliminary data analysis revealed subsets of candidate CREs with highly correlated chromatin activity and TF binding profiles, and we developed a method to combine these first prior to model building. This served two purposes pertinent to model development; a technical complexity, where multiple correlated predictors can pose problems for penalized methods and a biological perspective, where correlated regions could represent collaborative regulation and could possibly be interacting in three-dimensional chromatin structure. Two candidate CREs were linked in a network graph (Fig 1A, step 4) if they had high correlations in both their chromatin activity and TF binding profiles, and from this graph we obtained subsets of candidate CREs that form communities (dense sub-graphs), identifying these as coCREs (see Methods). The chromatin activity profile (CAP) of a coCRE is the mean of the CAPs of the community members. The coCREs along with the CREs near a gene were used to build a model predictive of the gene’s GEP. The models based on coCREs performed better than models with all CREs used separately, in terms of drop-in-variances (p ~ 10^−16^, see methods section) and model p-values (p ~ 10^−13^) (S3F Fig), and this methodology was retained for the remainder of the study.

### Gene specific predictive models

In order to generate gene-specific predictive models the candidate CREs within 100 kb of the TSS of a given gene were considered, and were reduced to a set of coCREs and singleton CREs (those not in any community). To affect gene expression, distal CREs such as enhancers need to interact with protein complexes nearer to the TSS, therefore singleton CREs were reduced to those proximal (within 20kB) to the TSS, limiting distal CREs to those that occur in communities (coCREs). The CAPs of this mixed set were considered as the initial predictors in the model building. Since the total number of candidate CREs mapped to genes are generally more than the number of cell types used (Fig 1B), we have used a penalized regression model (LASSO) [48, 49], which employs an additive penalty term with weight λ on the sum of the absolute sizes of the regression coefficients. Appropriate λ values were determined by cross-validation (Fig 1C) and two models were computed for each gene at λ = λ_*min*_ (minimum cross-validated mean square error) and at the more conservative (fewer non-zero regression coefficients) value obtained by adding one standard error λ = λ_1*se*_ [50]. The CREs or coCREs with non-zero regression coefficients β, were deemed to be the most predictive of the gene expression by the model (λ_1*se*_) and were termed ‘chosen CREs’ in this study. Statistical significance in LASSO models is an open area of research [48–51], since inference must account for the fact that the method sequentially chooses the most predictive variables from a set of candidates. We adopted the covariance test [51] as a means to give a *p* value to each non-zero coefficient in the model.

**Fig 2:**
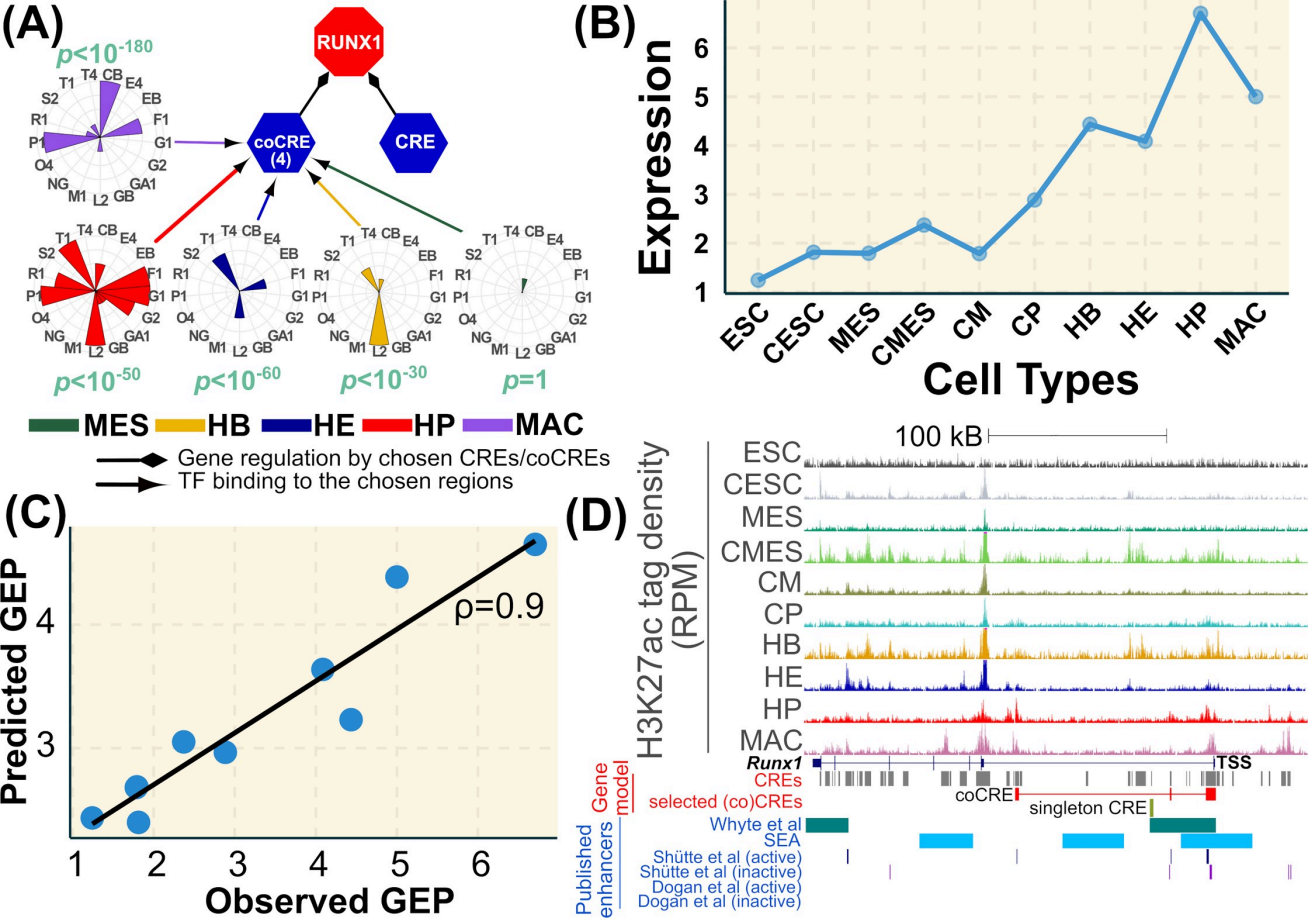
Predictive model for an example gene, Runx1. (A) A network representation of the model, where the gene (here *Runx1*) for which the model is built (red octagon), chosen CREs (blue hexagons) and TFs bound to the chosen CREs are represented as nodes. Black arrows indicate the regulation of the gene by the CRE/coCRE and coloured arrows represent the binding of TFs to the CREs in different cell types. The colours corresponding to the cell types are given below the network. The TFBP of the CRE in a specific cell type is represented as a circular histogram and in the case of coCREs these represent the frequency of occurrence of a specific TF in the regions of that community (here the community comprises of 4 regions). The *p*-value of observing a combinatorial binding profile in that cell type is provided for each TFBP node and the methodology is given in Methods section. The abbreviations for the TFs in the circular histogram are: Esrrb (EB), Nanog (NG), Pou5f1 (O4), Sox2 (S2), Cebpb (CB), Elk4 (E4), Gata2 (G2), Lmo2 (L2), Tal1 (T1), Fli1 (F1), Tead4 (T4), Meis1 (M1), Gata1 (GA1), Gfi1 (G1), Gfi1b (GB), Runx1 (R1), Spi1 (P1). It should be noted that not all TFs in the circular histogram have supporting ChIP-seq data in all cell types (Table 1). In the absence of ChIP-seq data for a specific cell type, the bar for that TF in the histogram of that cell type is zero. (B) The gene expression profile (GEP) of Runx1 with cell types along the horizontal axis and FPKM on the vertical axis. (C) The plot shows the best linear fit between the actual (X) and predicted (Y) GEP for Runx1. The spearman correlation coefficient is also provided. (D) The plot shows the tag density profile normalised as coverage per million aligned reads for the 10 cell types. *Runx1* gene structure is provided in blue below the coverage tracks. The predictor CREs that were used in the lasso model are given as grey boxes and the chosen CRE and the coCRE are given in red and yellow respectively. The super enhancers (SE) identified by Whyte et al.[53] are given as green bars and the enhancers given by SEA is in blue. The experimental enhancers identified by Schütte et al. and Dogan et al. are provided as well. In the case of *Runx1* there is no overlap with the Dogan et al. dataset, and hence the absence of any bars. It should be noted that the coCRE enhancer is represented as a composite of red boxes of member CREs.

An example of a predictive model for *Runx1* is given in Fig 2 (see Fig 1C for the cross-validation plot). *Runx1* is a gene encoding a transcription factor that is crucial for normal haematopoiesis [52] and is expressed during the later stages of the hematopoietic specification (Fig 2B). A set of 21 CREs and 3 coCREs were considered as the initial predictors, and two regions with non-zero β at λ_1*se*_ (Fig 2A and 2D) were selected as chosen CREs by the model. One of these is a coCRE, a community of four CREs bound by a range of blood specific TFs in different haemopoietic cell types (Fig 2A) and the other a singleton. The expression that is predicted by the model correlates well with the predicted gene expression profile for *Runx1* as shown in Fig 2C. The covariance test gives *p* values of 0.00 and 0.42 (S3 Table) for the chosen coCRE and singleton CRE above, showing strong evidence for a relationship between activity at the coCRE and gene expression.

Following the example of *Runx1*, models were generated for each member of the TF set. Full details can be found in Table 2 and S3 Table, and details of the chosen (co)CREs for the TF set are shown in S4 Fig. We chose to consider a model as potentially interesting if it had a least one non-zero β with adjusted Benjamini-Hochberg *q*-value based on the covariance test of < 0.05. Focusing first on the TF set, models were successfully built with at least one predicted (co)CRE for 14 of the 17 TFs and 9 of these were considered interesting by this statistical criterion.

**Table 2:** Generation of models.

Gene set	Number of genes	Number of models with at least one β≠0	Number statistically significant^1^	Number of coCREs with β≠0	Number of singleton CREs with β≠0
**TF set**	17	14 (82%)	9 (53%)	34	22
**TF cluster set**	437	340 (78%)	226 (52%)	163	282
**DE gene set**	9854	6715 (68%)	3212 (30%)	2592	5567

^1^A model is considered statistically significant if it has at least one regression coefficient β with Benjamini-Hochberg *q* value < 0.05. The details of the model parameters and the CREs are given in S3 Table.

Considering the TFs associated with pluripotency (see S4 Fig), *Nanog* is mapped to four singleton CREs and two coCREs, but only the coCREs are statistically significant. The coCREs are located on either side of the *Nanog* promoter with one coCRE (a community of 4) overlapping with its TSS and bound by all the four pluripotent TFs. *Pou5f1* that codes for OCT4 is mapped to one coCRE that includes the promoter region and a downstream region, and is bound to pluripotent TFs in ESC. Three CREs are mapped to the *Sox2* gene, two of which are coCREs comprising 2 and 3 regions respectively. One of the regions (the community of 3 CREs), although in this case not statistically significant, is almost 75kB away from the promoter, bound by the OCT4/NANOG/ESRRB TFs on all the three sites and was identified as a super-enhancer by Whyte et al. [53].

Regarding TFs specific to the hematopoietic lineage, we commented on the chosen CREs for *Runx1* above (Fig 2A). The coCRE is related to the +23kb region that is experimentally verified [54]. It is bound mostly by LMO2 in HB, TAL1 in HE and the LMO2/TAL1/FLI1/GATA1/PU.1 complex in HP and the increasing recruitment of TFs and the increasing significances of the combinatorial binding closely follows its expression profile (Fig 2A). *Tal1* has a single coCRE encompassing the promoter region and two downstream regions, separated by around 30kB [15]. The coCRE is primarily bound by LMO2/TAL1/GATA2/TEAD4 in HB, LMO2/TAL1/FLI1 in HE, FLI1/PU.1 in HP and PU.1 in macrophages (S4 Fig). *Gata2* has only one CRE mapped to it and it overlaps with its promoter region and is bound by LMO2/TAL1/FLI1 in HE and HP. It should be noted that although this locus fails to attain statistical significance (S3 Table) it is experimentally well-characterised [55]. *Lmo2* is mapped to a CRE lying on the proximal promoter [56] and the other coCRE bound predominantly by PU.1/CEBPβ in macrophages lying on the distal promoter region. *Spi1* (PU.1) is a macrophage specific TF that is mapped to two coCREs upstream of its TSS, with one coCRE overlapping with its promoter and is bound by LMO2/FLI1/ CEBPβ in HP and with LMO2 possibly replaced by PU.1 in MAC. This coCRE comprises of the promoter and a -17kB upstream region, which has been shown to be involved in *Spi1* expression control [28]. *Cebpb* is mapped to multiple CREs with one CRE mapped to the promoter region and a coCRE that spans around 50kB and is bound by PU.1/CEBPβ TFs. The coCRE is the most significant predictor and it overlaps with one of the super-enhancers upstream of *Cebpb* and with a region lying in an intron of the *Tmem189* gene downstream of *Cebpb* that shows high H3K27ac activity specifically in macrophages. This indicates the involvement of PU.1 or PU.1/CEBPβ complex in upregulating *Cebpb* in macrophages.

These results show that the proposed method has sufficient statistical power to discover at least one significant predictor, which is a potential regulatory element, for more than half (9/17) of this small set of key transcription factor genes. It is noteworthy that in many cases this element is a coCRE, suggesting that these CRE communities are more likely than singleton elements to be predictive of gene expression.

### Characteristics of the predicted cis-regulatory elements

**Fig 3:**
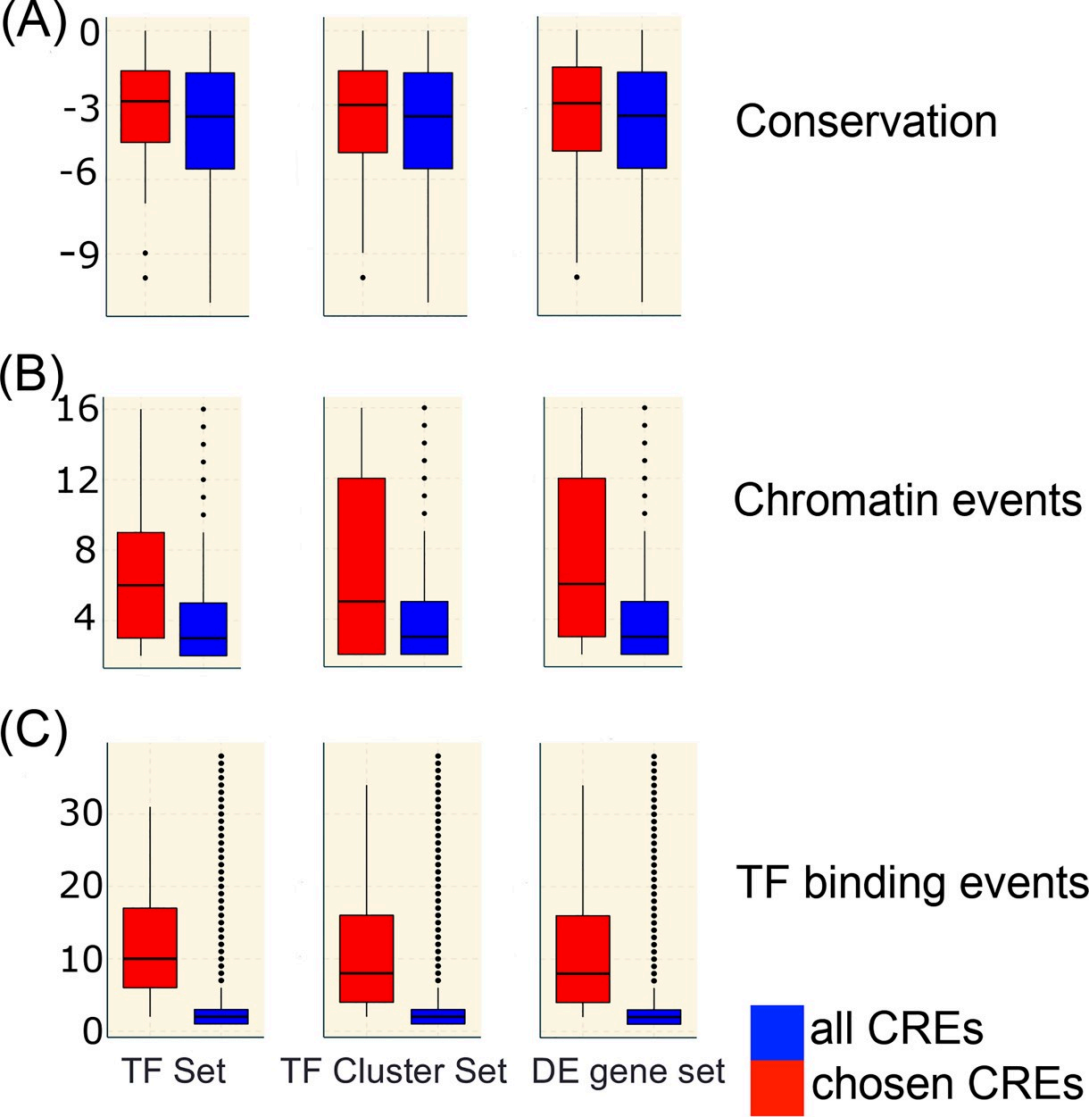
Characteristics of chosen regions. Conservation, chromatin events and TF binding events in chosen CREs (red) compared to all candidate CREs (blue). Left is the TF gene set, middle the TF cluster set and right all differentially expressed genes (DE set). (A) The log of the phylogenetic conservation scores (see Methods), (B) the chromatin events (H3K27Ac peaks and DHS), and (C) TF binding events. The *p* values were obtained using student t-test for conservation and Kolmogorov-Smirnoff test for chromatin and binding events. All the *p* values were less than 0.01 except for conservation distribution in TF set (*p* = 0.07).

The TF cluster and the DE gene sets represent much larger sets and the data in Table 2 show that the method still generates a useful proportion of statistically significant models, although this falls to 30% of genes in the largest set. Full details of the models are in S3 Table. Fig 3 shows an analysis of the characteristics of the chosen CREs within statistically significant models. The phylogenetic conservation scores of the chosen CREs are significantly high compared to the score distribution of the all CREs (Fig 3A). Similarly, the occurrences of H3K27ac peaks and DHS across all cell types are higher for chosen CREs (Fig 3B) as are TF binding events (Fig 3C). These observations suggest that regions that are predictive of expression are hotspots for epigenomic activities and TF occupancies, confirming earlier observations [57].

### Selected regulatory regions as enhancers

**Fig 4:**
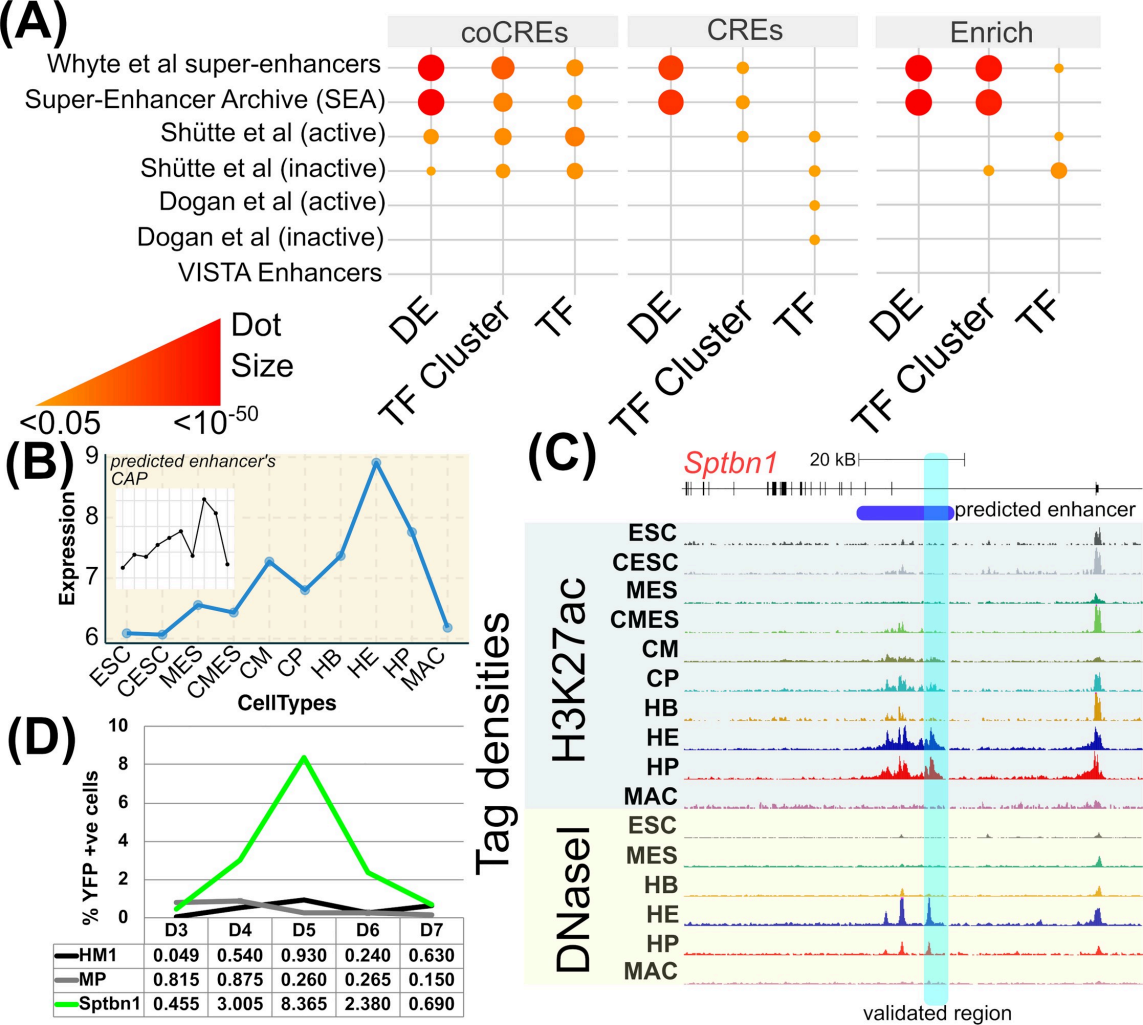
Validation of predicted enhancers. (A) Overlap of genomic regions between published sets of enhancers (vertical axis) and the CREs/coCREs chosen for genes in the three gene sets (horizontal axis). The dot plot indicates the significance (-log_10_(*p*) with *p* adjusted for multiple testing) of the pairwise overlaps (red/large size = high significance, orange/small size = low significance). The absence of a dot signifies *p* > 0.05. The right panel (Enrich) shows a negative control of overlaps with candidate CREs that were not chosen as predictive by our method but with H3K27ac enrichment level similar to the chosen (co)CREs. (B) Expression of *Sptbn1* across stages of haematopoietic and cardiac differentiation shown as a blue line chart. The inset plot shows the chromatin activity profile (CAP) for a CRE predicted to be associated with this gene. (C) A UCSC browser snapshot of the predicted CRE within the Sptbn1 gene body. The snapshot shows this region shaded in blue, illustrating the dynamics of the active chromatin mark, H3K27ac (top), and chromatin accessibility (bottom) across the cell types. The coordinate considered for further validation is chr11:30166167–30166587 highlighted in transparent cyan box. (D) A 5-day time course of haematopoietic differentiation, tracking the expression of a YFP reporter gene driven by the predicted CRE. Expression peaks on day 5 (D5), which is equivalent to the haemogenic endothelium (HE). The controls are the ESC line HM1 (black) and HM1 cells targeted with the reporter construct containing the minimal promoter (MP) only (grey).

It is to be expected that a substantial number of the chosen (co)CREs are enhancers or include enhancers as sub-regions. In order to investigate this further, we considered existing datasets of enhancers from disparate sources including a dataset validated using *in-vivo* screening (VISTA) [58], two sets that were collated based on TF binding and further experimental validation (Schütte et al. and Dogan et al.) [22, 24], a set of super-enhancers identified using genome-wide binding profiles of TFs along with Mediator (Whyte et al) [53] and a dataset generated through integrative selection from various different NGS resources (SEA) [59]. It is to be noted that any comparison is likely to be affected considerably by the type of data used for integration in genome-wide enhancer datasets, the type of cells used in experimental validation of enhancer activity, and the experimental or computational methodology used by the authors in compiling the dataset. In this analysis, we have therefore attempted a conservative and unbiased approach of studying genomic overlaps between the chosen CREs/coCREs with each of the known enhancer datasets. The results are shown in Fig 4A and the total number of regions that overlap is provided in S4 Table. As expected there is a significant overlap with genome-wide methods (Whyte et al. and SEA) that follow a similar approach. The overlap with Schütte et al. is high but the selected CREs show no significant overlap with datasets by Dogan et al. and VISTA. The likely reason for this discordance is the fact that Schütte et al. examined enhancers active in haemopoietic stem/progenitor cells which will be similar to the enhancers activated during haemopoietic specification (our study). Furthermore, with reductionist approaches the analysis is influenced by the genes studied in the experiments. For example, VISTA database has only 0 (0%), 6 (1%) and 75 (14.5%) of genes that overlap with our TF, TF Cluster and DE gene sets respectively. As a negative control, we chose to compare the overlaps of candidate CRE regions with similar H3K27ac levels that were not chosen as predictive by our method (Fig 4A, right hand panel). As expected, genome-wide data sets (SEA and Whyte) had significant overlaps with these negative control CREs. However, in the cell type specific data set of Schütte et al. we found that these negative control regions overlap less significantly with active elements and more significantly with inactive elements.

We performed an in-depth gene-wise analysis of the overlap with Schütte et al. because of the significance of genomic overlap and also the similarity in the cell types used provides us with an ideal, albeit small, dataset to check if the chosen CREs’ roles are enhancers (details in S4 Table). The dataset overlaps with enhancers of 9 genes with 5 of the lasso models having a q value ≤ 0.05. The coCRE predicted by our *Runx1* specific model overlaps with 3 active and 1 inactive enhancers by Schütte et al. (Fig 2D). Only one active enhancer (Runx1+204 [24]) was rejected by our model because of the 100kB distance cut-off (see Methods). In *Meis1* the singleton chosen CRE (S3 Table) overlaps with the only active +48kB hematopoietic enhancer identified by Schütte et al. The coCRE of the *Spi1* (PU.1) overlaps with one enhancer and one inactive region, where the latter is a promoter region (S5A Fig). The coCRE of Tal1 overlaps with all the six enhancers (3 active and 3 inactive) from Schütte et al. (S5B Fig). Interestingly one active enhancer was correctly mapped to the coCRE of *Tal1* although the CRE is in close proximity with the neighbouring gene, *Pdzk1ip1*. Although this enhancer is located far away from the other active enhancers, being part of a single coCRE may be an indication of interaction. In *Erg*, the model was able to map all the active enhancers to the single chosen coCRE (S5C Fig). In *Lyl1* the chosen CRE extends over a broad region comprising of one active and two inactive enhancers. However, in the case of *Fli1* the singleton CRE overlapped with two haemopoietically inactive elements.

While our method is intended to produce gene specific regulatory models, and does not aim directly to predict enhancer-promoter interactions, we considered that it would be useful to compare the information from our model with that from a contemporary method for the latter problem. We trained the JEME method [40] on 3D interaction data from ESC cells with the reduced feature set available in our data, and used this to predict interactions in other cell types (see Methods), focusing here on predictions in HP cells. An example of the *Runx1* gene is shown in S5D Fig and summary results over all nine Schutte et al. genes are in Table 3 and S5E Fig. In the case of *Runx1*, our retrained JEME predicts 10 potential enhancers within 100kB interacting with the promoter; these overlap all four identified as positive by Schutte et al., and only one of the four identified negatives. The remaining six JEME predictions represent untested regions, and may be false positives but equally could be regulatory. Our method on the other hand makes only five predictions, which overlap 3/4 positives and 2/4 negatives. Results for other genes are similar (Table 3 and full detail in S5 Table). Overall the retrained JEME makes more predictions (S5E Fig), some of which may be false positives, and over the tested regions it is slightly more accurate than our method, although the difference is not statistically significant.

**Table 3:** Overlaps of predicted regulatory elements with experimentally tested regions.

Gene	Predictions^1^		Overlap +ve^2^		Overlap–ve^3^		No overlap^4^	
	JEME	CRE	JEME	CRE	JEME	CRE	JEME	CRE
*Erg*	12	4	1/3	3/3	1/2	0/2	10	2
*Fli*	15	1	2/2	0/2	0/3	2/3	13	0
*Gata2*	7	1	2/4	2/4	0/2	0/2	6	0
*Gfi1b*	15	4	3/3	0/3	1/1	0/1	11	4
*Lyl1*	13	4	1/1	1/1	1/2	2/2	12	3
*Meis1*	10	1	1/1	1/1	2/3	0/3	6	0
*Spi1*	14	4	0/1	1/1	0/2	1/2	14	2
*Runx1*	10	5	4/4	3/4	1/4	2/4	6	1
*Tal1*	10	3	2/3	3/3	0/3	3/3	8	0
**Totals**	**106**	**27**	**16/22**	**14/22**	**6/22**	**10/22**	**86**	**12**

1. The number of predicted regulatory regions within 100kB of the TSS for each method. (The present method is indicated as CRE in the table)

2. ‘True positives’–the number of regulatory regions experimentally verified as positive and overlapped by a predicted region. Numbers in the denominator indicate the number of experimentally verified positive regions for this gene.

3. ‘False positives’–the number of regions experimentally verified as negative and overlapped by a predicted region. Numbers in the denominator indicate the number of experimentally verified negative regions for this gene.

4. The number of predicted regions that do not overlap experimentally tested regions. Either false positives or novel discoveries.

### Novel enhancers for *Sptbn1* and *Nfe2* genes verified experimentally

We next sought to assess the utility of our gene specific chosen CREs by experimental testing of previously unknown potential enhancer elements contained within them. We selected two of our predicted CREs that exhibit discrete CAPs and that are associated with genes that are important for haematopoiesis, namely *Nfe2* and *Sptbn1*. *Nfe2* encodes for NF-E2 transcription factor expressed in HSCs, erythroid, myeloid and megakaryocytic lineages, and is known to be involved in epigenetic modification thereby regulating blood cell maturation programmes. Abnormal expression of this gene is linked to pathogenesis of myeloproliferative neoplasms (MPNs) [60]. *Sptbn1* encodes for β spectrin, a cytoskeletal protein and has been implicated in the determination of cell shape, organelle organisation and cellular traffic [61]. A fusion gene SPTBN1-FLT3 has been observed in a small population of BCR-ABL-negative CML [62].

For both of these genes the CREs were obtained from their respective gene-specific models using λ_1*se*_. We segmented the predicted CRE region into 500 bp windows and identified the window with the highest number of TF binding sites within it. This TF hotspot within the predicted CRE was then cloned upstream of a reporter gene and used for single site targeted integration into a mouse ESC line as described previously by Wilkinson et al. [63]. This enabled us to follow the dynamics of reporter gene expression during blood specification.

Out of 11 possible predictors for CREs associated with *Sptbn1*, one is chosen within the gene body and it is evident that its H3K27ac and DHS profile across the cell types coincides with the expression profile of the gene (Fig 4B and 4C). The TF hotspot for this CRE shows binding of TEAD4, TAL1, LMO2 in HB, FLI1, TAL1, LMO2 in HE and RUNX1, GFI1, GATA2, TAL1, LMO2 in HP to this 500bp region (highlighted by cyan box in Fig 4C). Consistent with our CRE prediction, we see enhancer activity with the reporter gene assay (Fig 4D) and this shows an expression profile that reflects profile in Fig 4B with high expression in HE and HP cells. In the case of *Nfe2* (see S6 Fig) we find two predicted CREs and this presents an interesting case because the q value of the model unlike *Sptbn1* is high (S3 Table). CRE1 shows a promoter like profile, having a high H3K27ac peak showing a characteristic bimodal distribution that dips where there is enrichment for hypersensitive sites (S6 Fig). CRE2 presents an interesting case because it does not have a strong H3K27ac signal, but has a very specific DHS at the HB stage that progressively opens up until HP. Also, the CAP correlates well with the GEP of *Nfe2*. Furthermore, its TF hot spot is bound by the LMO2, TAL1 complex from HB to MAC and is joined by the binding of GATA1, GATA2, GFI1 and GFI1B in HP where the chromatin activity is the highest. The reporter gene assay shows a very similar expression profile to the CAP, with expression as early as HB, peaking on day 5 when HPs begin to emerge. Therefore, both enhancer studies demonstrate that the dynamics of expression regulated by the predicted CREs follows the CAP used in the model building.

### Combinatorial binding and transcriptional regulatory network

**Fig 5:**
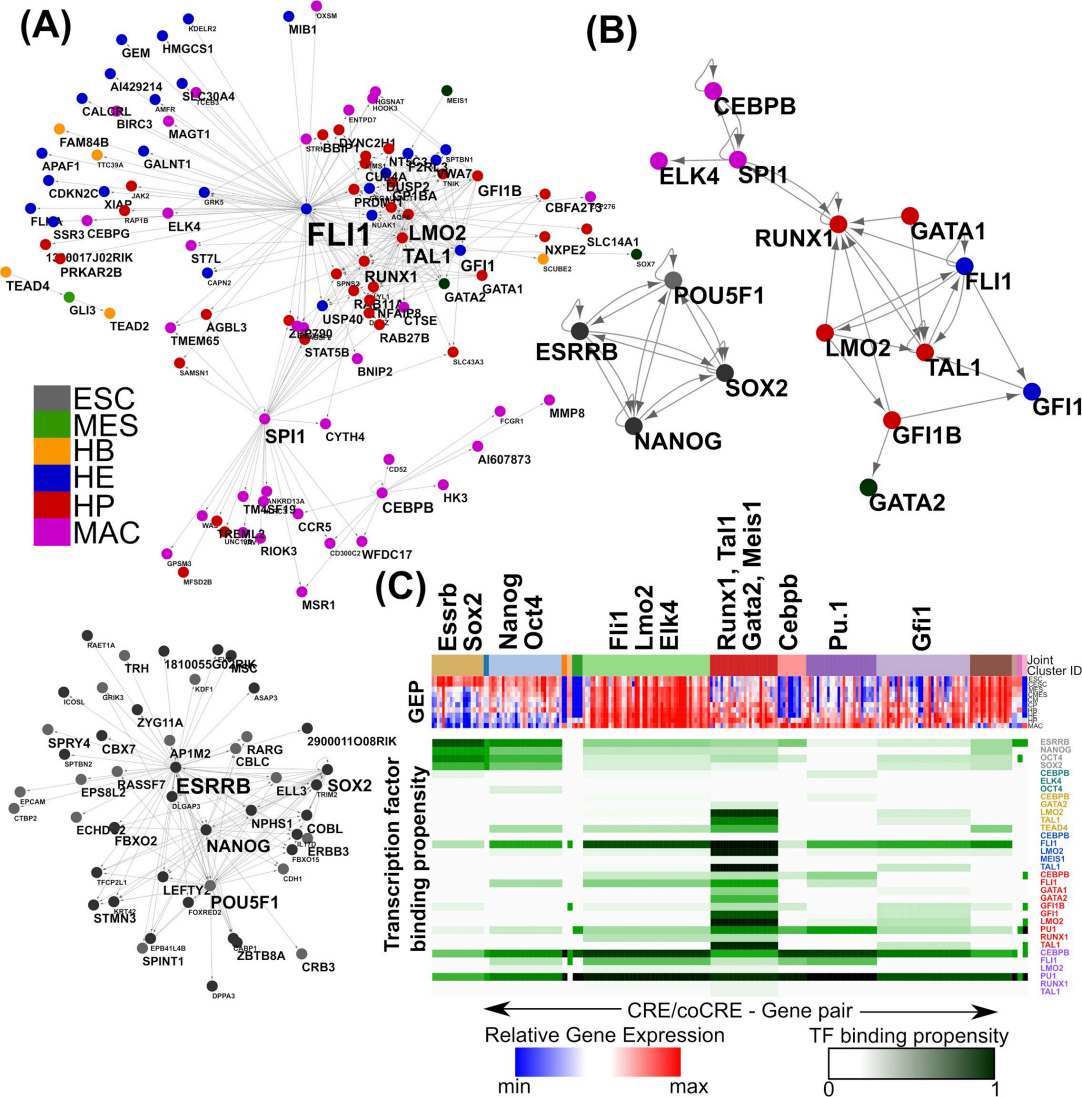
Cis regulatory networks (CRNs) and joint clustering of expression and regulation. (A) CRN for the TF cluster set. The genes are represented as nodes and directed edges show the genes that are co-expressed and also bound by the TF of one gene (source) to a predicted CRE of the other gene (target). The cell type at which the expression of the gene is highest is shown as colours on the node. The size of the node name is proportional to its degree. (B) The CRN for the TF set (colours as A). (C) Joint clustering of genes in the TF cluster set. Genes cluster together according to the relatedness of both gene expression patterns (red-blue heatmap) and the binary pattern of TF binding (green-white heatmap) at their main predicted (co)CRE. Each cluster is distinguished by a colour coded bar above the GEP heatmap (highlighted as “Joint cluster ID”). For each cluster, the average of the TF binding profile is shown as the TF binding propensity, where 0 represents absence of TF binding and 1 represents binding of that specific TF in all the regions belonging to that cluster (green = TF binding; white = no TF binding).

The complex structure of transcriptional regulatory networks in higher eukaryotes reflects the combinatorial control of genes by multiple transcription factors. It is poorly understood owing to the multiplicity of potential regulatory elements and the associated difficulty in assigning distal elements to the genes they control. The methods introduced here allow the construction of networks where transcription factors link only to genes for which they bind to chosen CREs and with which they are co-expressed. These networks contain putative causal regulatory relationships and here are called cis regulatory networks (CRNs); they are approximate and likely to reflect only the strongest aspects of control for each gene, but nevertheless they represent a substantial simplification compared to alternative approaches. S7 Fig illustrates this in comparison to simple co-expression networks (CENs, where TFs are connected to all genes whose expression is correlated). This shows that the CRN is simpler in terms of vertex degree and connectivity. It is also clear that those TFs with high betweeness centrality, a measure of the importance of the node to the overall network structure, stand out much more clearly in the CRN, and are enriched for known critical haemopoietic transcription factors, TAL1, RUNX1 and FLI1. Fig 5 illustrates CRNs for the TF cluster set (Fig 5A) and the TF set (Fig 5B). It is evident that the regulation involved in maintaining pluripotency is disconnected with other systems and that there are at least four different sub-graphs of regulation: (i) the pluripotency network controlled by Nanog, Oct4, Sox2 and Esrrb, (ii) the HE dominant network controlled by Fli1, (iii) the HP network comprising of the Runx1, Scl/Tal1-Lmo2 TFs and (iv) the MAC specific network with Pu.1 (Spi1) forming the nexus between the HE/HP networks and the Cebpb network.

Network complexity in higher eukaryotes has so far made it difficult to answer the fundamental question of how many different genes are regulated in the same way, or if a similar pattern of gene expression can be produced by different regulatory processes. Fig 5C shows a preliminary analysis of this question where genes are assigned to clusters where they share common patterns of both gene expression (GEP) and transcription factor binding (TFBP) to their single most significant/predictive (co)CRE, using a recently introduced joint clustering method [64] (see Methods section). This analysis uses the “*TF cluster set*” with genes having chosen (co)CREs bound to only one TF binding event removed, reducing the gene set from 437 to 222 genes, in order to focus on genes with clear combinatorial TF binding.

Clusters obtained from joint clustering are rather different to those from “expression only” clustering shown in S2B Fig. Clusters based on joint information (TF binding and gene expression) are approximately two times larger than those based on gene expression only, and represent a broader range of expression patterns: in the case of using expression information only, most of the TFs lie in separate clusters (with the exception of Tal1 and Gfi1b), while including TF binding information draws together TFs into shared clusters. This suggests that shared TF regulation leads to a broad range of similar gene expression profiles, and that other regulatory processes, beyond the information in this data set, could define the detailed differences shown between the smaller “expression only” gene clusters.

Among the interesting aspects of biology suggested by joint clustering is that the pluripotency factors Esrrb and Sox2 form a separate cluster to Nanog and Oct4, which show an expression pattern that persists longer and is associated to different TF binding profiles. In relation to haematopoiesis, genes in the cluster containing Fli1, Lmo2 and Elk4 are bound predominantly by FLI1 at their CREs in HE and HP suggesting a tightly controlled regulatory mechanism by Fli1 underlining its importance in haemopoiesis and vasculogenesis [65]. Genes in the cluster with Runx1 express in the later stages of haemopoiesis (HE, HP, and MAC) and exhibit a more complex binding pattern of factors including FLI1, SCL/TAL1, RUNX1 and GATA2 in HB, HE and HP. Thus, the hematopoietic differentiation from HB to MAC seems to be regulated by distinct regulatory mechanisms at various stages of the pathway that start with the FLI1 controlled genes co-expressing with Fli1 and Lmo2 in HB resulting in the initial activation of hematopoietic genes, followed by the controlled cluster of Runx1, Tal1, Gata2 and Meis1 involved in the formation of HE and later the transition from HE to HP dictated by CEBP/β bound genes and finally PU.1 specifying the macrophage transcriptional network.

### Conclusion

Transcriptional regulation can be investigated at the level of a single gene, where studies lead to detailed understanding of all or most relevant cis-control elements, or at genome-scale where high-throughput studies can reveal many general aspects of regulation. Here we have attempted to create methodology that can bridge the gap between these contrasting types of study. One of the challenges of genome-scale study is that the data sets involved are indeed very large, but the information per gene is relatively small. Coupled with the plethora of potential regulatory elements for any gene in higher eukaryotes, it is a significant challenge to identify those elements most relevant to its regulation. We have shown that careful integration of high-throughput data of a range of types and sources, coupled with the building of gene specific predictive models, can identify a few (typically 1–4) statistically significant potential regulatory elements for a large proportion of genes. We view the method as a means of hypothesis generation that will feed into further detailed study of individual genes and regulatory elements, and here we have illustrated this with experimental confirmation for two example genes.

The CREs identified by our method, show many of the expected characteristics, including higher than average conservation, binding of transcription factors and activity in multiple cell types. Counter-point to this is that many of the CREs are not conserved, perhaps reflecting the fact that regulation of genes may diverge more quickly than the genes themselves in closely related species, showing that conservation is not an effective way of reducing the number of candidate CREs in model building. Introducing the concept of a community of CREs, related by similar profiles of transcription factor binding and activity measures across the cell types involved, has aided this work technically by reducing the number of potentially predictive variables and reducing multicolinearity. Equally importantly, it may have deeper biological significance, for instance in three-dimensional genome structure, and this will be a subject of future investigations. This feature of using network-based construction of collaborating CREs prior to model building, to the best of our knowledge, is presented for the first time in this work. From our analysis of haemopoietic master regulators, we found that many of the chosen coCREs contain elements close to promoters, but also link in distal CREs (≥20 kB) with similar profiles of transcription factor binding and activity.

In comparison of regulatory regions chosen by the models with experimentally and computationally known enhancers we find that coCREs overlap more significantly than CREs, further emphasising that collaborations between distal regulatory elements could be a prevalent mode of transcriptional regulation. In addition, we were able to exploit the loci identified by the models to transform incomprehensible co-expression networks (CENs) to tangible cis-regulatory networks (CRNs). The derived CRNs more clearly define the networks and subnetworks of TFs and master regulators responsible for different stages of differentiation.

The comparison of our method with JEME provides an interesting perspective on two completely different computational approaches to predicting how genes are regulated, one (ours) designed to be independent of 3D chromosome interaction data and to identify a small number of the most predictive elements for the expression of a gene, and the other trained on, and designed to predict 3D interactions. Comparison of methods in this area for mouse is difficult owing to a lack of gold standard data for real regulatory relationships. Three-dimensional interaction is often used in this way, but not all interactions are regulatory [66]. Comparison on the Schutte et al. data set, albeit relatively small, showed the methods to have similar performance. The JEME related method produced more predicted elements for each gene and may have a higher false positive rate for this reason. On the other hand, for known elements, performance of our method was marginally worse, but this difference was not statistically significant. Overall these results show that our rather simple method, based on the limited data sets available for mouse cells in this case, is useful and able to perform comparably to a method developed for the larger data sets typically available for human cell lines.

## Materials and methods

### Dataset

For this study, high-throughput data from two *in vitro* differentiation pathways of mouse embryonic stem cells (ESCs) were considered: differentiation to macrophages [15] and to cardiomyocytes [47]. The fastq files for H3K27ac, DNaseI, RNA-seq and Transcription Factor (TFs) experiments were downloaded from Gene Expression Omnibus (GEO identifiers: GSE69101 and GSE47950 respectively). Gene and transcript definitions from RefSeq [67] were obtained using the UCSC Table Browser utility [68, 69]. The conservation scores are the phastCons evolutionary conservation scores for 60 vertebrate species obtained from the UCSC genome browser using its Table Browser. Enhancers defined by Whyte et al. [53], Schütte et al. [24] and Dogan et al. [22] were obtained from their respective publications. The enhancers from SEA [59] and VISTA [58] were downloaded from their respective websites.

### Data processing

The sequences were processed uniformly by first trimming reads for sequence quality of 20 using Cutadapt [70]. The trimmed reads were aligned to mm10 (UCSC genome browser) using Bowtie2 [71] for letter-space reads and SHRiMP [72] for colour-space reads. Only reads aligned to unique chromosomal positions and with a mapping quality of at least 20 were retained for further calculations. Total reads that overlap the exons of the genes were calculated using HTSeq-count [73]. Gene level expression values were computed as reads per kilobase of exons per million mapped reads (RPKMs) [74] (Eq 1), and a standardised expression value for a gene was computed as (*x*-*μ*)/*σ* where *x* is the expression value, *μ* the mean and *σ* the standard deviation over cell types. For the initial data analysis, the expression data for protein coding genes in each cell type were analysed by principal component analysis (PCA) and hierarchical clustering to confirm the expected biological relationships between the cell types (see S1 Fig).

$$RPKM=\frac{n\times 10^{9}}{s\times N}$$ (1)

Here *n* = total number of reads aligned to exons of size *s* bp and *N* is the total reads aligned to the genome.

DNaseI Hypersensitive Sites (DHSs) were called using DFilter [75] with default parameters (-bs = 100 -ks = 50 –refine and FDR ≤ 0.05) while the TF peaks were called using MACS (default parameters) since the method of generating shifting tags is well suited for DNA binding proteins. For TF peaks manual assessed *p*-values were used and are described in Goode et al. [15]. H3K27ac enriched regions in hematopoietic lineage were the “gapped peaks” called by MACS2 [76] using *q*-value cutoffs of 0.01 for narrow peaks and 0.1 for broad peaks [39] and the H3K27ac enriched regions in the cardiac lineage were obtained from Wamstad et al. [47]. DHSs and H3K27ac peaks that had an overlap of at least 125 bps were merged and the read abundances in these peak regions were computed as RPKMs (Eq 1, where *s* is the size of the region in bp) using BedTools [77]. Conservation scores for the regions were obtained as the 3^rd^ quartile value (top 75%) using bwtool [78].

Differentially expressed genes were identified by all against all pairwise cell type comparisons using DESeq [73]. A gene was defined as differentially expressed (DE) if its expression has changed at least four-fold with an adjusted *p*-value of less than 0.05 in at least one comparison. We identified 9854 genes that were differentially expressed and with expression value greater than 1 in at least one cell type. The standardised expression values of these DE genes were then clustered using *k*-means and the Bayesian Information Criterion (BIC) scores were obtained as a function of *k* using the adegenet R package [79, 80]. The optimal number of clusters (*K* = 391) was thus determined as the *k* with the lowest BIC score (S1D Fig). For a given DE gene its Gene expression profile (GEP) is an *m*-dimensional vector where m is this the total number of cell types analysed (here *m* = 10) standardized gene expression values over all cell types, GEPs) were generated for all the differentially expressed genes considered in this study.

### Identification of candidate *cis* regulatory elements (CREs)

The DHS and H3K27ac peaks that had an overlap of at least 125 bps were merged to obtain the initial set of candidate *cis*-regulatory elements (candidate CREs). For each candidate CRE, a “chromatin activity profile” (CAP) is a *m*-dimensional vector representing the chromatin activity of the CRE in the *m* cell types (here *m* = 10). For a given CRE and a given cell line the chromatin activity is the log_2_(RPKM) of H3K27Ac computed as given in Eq 1 where *s* here is the width of the H3K27ac peak, and if DNaseI-seq data was available for the cell type, the average of the log_2_RPKM of the H3K27ac and the DNaseI-seq data (Table 1), since these two measures have been shown to be highly correlated in cell type specific DHSs [81]. For each gene the predictors are the set of candidate CREs within 100kb of a gene’s transcription start site (TSS) identified using ChIPpeakAnno [82].

### Identification of community CREs

The CREs were overlapped with the 33 TF ChIP-seq peak sets (Table 1) and for each region the “TF binding profile” (TFBP) was generated as a binary vector indicating the overlap (1) or non-overlap (0) of the 33 peak sets (Fig 1A, Step: 3). For a given gene, we identified the *N* CREs with 100 kB of the gene and in order to generate community CREs (coCREs) two matrices of *N*×*N* dimensions were generated: (i) the activity correlation matrix (*C*) that represents the pairwise similarity between the CAPs of the *N* CREs, and (ii) the TFBP correlation matrix (*B*) that represents the pairwise similarity of the TFBPs of the *N* CREs. Each element of matrix *C* (*C*_*ij*_) is the Spearman correlation coefficient of the CAPs of the two CREs (*i* and *j*) and similarly *B*_*ij*_ is the Jaccard index of the TF binding profiles of *i* and *j*. From these two matrices, an adjacency matrix (*A*) of *N*×*N* dimensions were computed as given in Eq 2. A network then is a representation of *A* where CREs within 100kb of the gene represent the nodes and an edge between two nodes (if *A*_*ij*_ = 1) represent highly correlated activity of both their CAPs and TFBPs.

$$A_{ij}=\left\{ \begin{array}{l} 1\;\mathrm{if}\;B_{ij}{.C}_{ij}>0.5,\\ 0\;\mathrm{otherwise}. \end{array}\right.$$ (2)

For such a network, communities are the dense sub-networks of sets of nodes with relatively large numbers of edges among them and were identified using a greedy optimization of modularity score [83]. The coCREs are defined as communities of more than one region with at least two TFs binding to them. The CAP of a coCRE is the average of the activity profiles of the constituent CREs. To differentiate the rest from coCREs, candidate CREs not assigned to communities are referred to as ‘singleton CREs’.

### Penalized linear models

All coCREs within 100kB of a transcript’s start site (TSS), and singleton CREs within 20kb, were considered as potential predictor variables in building the linear model. The CAPs of the CREs were considered as a column in the independent term matrix (*X*). The response vector *Y*, consisting of expression values corresponding to the cell type in *X* was modelled using linear regression as

$$y=\beta_{0}+\sum_{i=1}^{n}\beta_{i}x_{i},$$ (3)

where *β*_*i*_ are the regression coefficients. For a penalized linear model, the aim is to minimize the penalized likelihood (assuming a normal distribution), ${min}_{\beta }({{\left\|y-\hat{y}\right\|}_{2}}^{2}+\lambda \sum_{i=1}^{n}\left|\beta_{i}\right|)$, where the penalty is on the summed absolute values of the regression coefficients and λ is the regularisation parameter. The *l*_1_ norm form of the penalty leads to zero values for a subset of the β coefficients, depending on the size of this parameter. A leave-one-out cross validation was performed for a sequence of λ values to obtain the optimal value of λ where mean square error is minimized λ_*min*_ and also λ_1*se*_ = λ_*min*_+1×*stdev*, which is a more conservative estimate for λ (Fig 1C). The CREs/coCREs with β ≠ 0 at the optimal λ are the most predictive for that gene, and are termed ‘chosen CREs’. If at the λ cutoff the total number of predictors with non-zero regression coefficients is zero, then the highest λ value at which at least one predictor has a non-zero coefficient was considered. The penalized regressions and cross-validations were performed using glmnet package in R [84]. In order to generate *p* values for the predictors with *β* ≠ 0 we have used the covariance testing as implemented by the covTest package in R [51] and adjusted for multiple testing using Benjamini-Hochberg method (*q* values). The covariance test tests the significance of a predictor as it enters the active set at a given step in the lasso path [51]. The *p*-value for a predictor takes into account the adaptive nature of lasso and also the shrinkage effects due to the *l_1_*-norm penalty. The *q*-value for each gene model is the *q*-value of the most significant predictor at λ_1*se*_. The models generated, the λ values, the chosen predictors along with the *q*-values are provided in S3 Table. It can be seen from S3 Table and S8 Fig that for the selected models there are non-zero beta with *p* > 0.05. They should be viewed with caution in comparison to better supported interactions, because on one hand the cross validation indicates they have a significant contribution to prediction accuracy but one the other hand the covariance test, which is more conservative, considers them insignificant.

Apart from using covariance tests for assessing a model’s significance one can obtain empirical *p*-values by randomising either the CAP or GEP. Here for a given gene, we retained the CAP of the predictors and randomised the GEP *N* = 10000 times and generating *N* models. The spearman’s correlation (*C*_*r*_) between the randomised GEP and the GEP predicted by the model is calculated. If the correlation derived for the actual model is *C*_*t*_, and *x* the total number of iterations where *C*_*r*_ ≥ *C*_*t*_, then an empirical *p*-value can be generated as *x*/*N*. The comparison between the two tests for model significance is given in S7E Fig. Although there is general agreement with the exception of Runx1 and Spi1, the randomisation approach is time consuming (limited by the number of iterations), and we preferred the covariance test method which is explicitly designed for LASSO models and therefore likely to be more powerful.

It should be noted that if there are more than one highly correlated CREs for a given gene that are predictive of the expression, then LASSO is designed to select one of the CREs. In order to ascertain the ability of the model to accurately choose the best correlated regions, we removed the chosen regions from the list of predictors for a given gene and generated a new model. We performed this analysis for all the genes in the three gene sets and found only 15 new models with at least one chosen region (*β* ≠ 0) at λ_1*se*_ and a *p*-value of less than 0.05. While this may remain an issue with LASSO regression, it indeed shows that in this study such effects are minimal possibly due to the construction of coCRE prior to model building.

For each gene, two models (λ_1*se*_) were generated, one with regions before the construction (pre-coCRE) and the other with regions after the construction of community CREs (post-coCRE), as predictors. The difference in drop-in-variances (Δ*div* = *div*(post-coCRE)–*div*(pre-coCRE)) and in *p*-values (Δ*pv* = *pv*(post-coCRE)–*pv*(pre-coCRE)) for the best predictor (highest *div*), were computed for any given gene if *p* < 0.05 in at least one of the two models. In order to ascertain the technical impact of coCREs, we used paired Student t-tests for computing the significances for higher drop in variances (+Δ*div*, S3F Fig, red), and decrease in *p*-values (-Δ*pv*. S3F Fig, red) in post-coCRE models compared to pre-coCRE models. S3F Fig shows that in the *TF Cluster* set, both the *div*, and *p*-values for post-coCRE models are significantly better than pre-coCRE models. This trend remains true when applied to all the genes considered in this study.

### Overlaps with data sets of experimentally determined enhancers

We considered the overlap of the set of (co)CREs chosen in our models with several external data set of enhancers described above (see Fig 4A), using the hypergeometric test. To form a negative control set for these studies, for each gene, a set of CREs/coCREs were selected from the available set of rejected predictors (*P*) that match the chromatin activity level of the predictors chosen by the model (*C*). If CAP_r_ is the chromatin activity profile vector of a CRE/coCRE (*r*), then *m*_*r*_ = max(CAP_r_). The set of rejected regions with matching chromatin activity for that gene is {*r*∈*P*|*m*_*r*_>*m*_*c*_−0.5} where *m*_*c*_ is the max(CAP) of any of the chosen predictors. Such sets were generated for the three gene sets (Table 2) and their overlaps with known enhancers are given in Fig 4A.

### Application of JEME to the myeloid lineage data set

Cao *et al*.’s Joint Effects of Multiple Enhancers (JEME) method employs a two-step machine learning framework to predict cell line specific enhancer-TSS interactions [40]. Firstly, LASSO linear regression models calculate the ability of DNase, H3K27ac, H3K27me3 and H3K4me1 signals to predict the expression of a TSS within 1Mb of an enhancer. Secondly, LASSO error terms are input, along with DNase and histone enhancer, promoter and window features, into a Random Forest classifier trained on chromosome conformation data.

JEME was applied to the myeloid lineage (ESC to MAC) dataset, for which DNase-seq, ChIP-seq and RNA-seq data were available. As we did not have data for H3K4me1, this mark was substituted for H3K4me3. Input files were processed as described in Cao et al. 2015 [40]. TSS co-ordinates were obtained from the mm10 RefSeq curated annotation using the UCSC Table Browser. Cell specific active enhancers were defined by the 4-state ChromHMM predictions from Goode *et al*. 2016 [15]. JEME code for Cao et al.’s ‘Roadmap + ENCODE’ dataset, was downloaded from https://github.com/yiplabcuhk/JEME and adapted for our dataset. JEME’s Random Forest classifier was retrained in WEKA [85] on a set of 18,353 positive and 122,353 background pairs, assembled from the ESC HiC data (GEO identifier: GSM2026260) [86] using the ‘random targets’ method [40]. Predictions were made for enhancer-TSS interactions in MES, HB, HE, HP and MAC cells, using the default threshold of 0.35. JEME predicted 64.2% of selected CRE-gene pairs, accounting for 7.9% of all JEME predictions within 100kB of a TSS considered by our method (p value for overlap ≪ 10^−65^). The results shown in Table 3 do not change significantly by varying the threshold.

### Joint clustering of TFBPs and GEPs

In order to jointly cluster gene expression and regulatory input we employed a method we recently developed [64]. This adopts a probabilistic mixture model-based clustering with integration of binary (TF binding) and continuous (expression) data using Bernoulli and Gaussian distributions respectively. The mixture components (clusters) represent sets of genes with related TF binding and expression patterns. In this context, a TF binding pattern is a binary string reflecting binding (1) or not (0) for a set of TFs to the chosen (co)CRE in question in each cell type. A data matrix was constructed with the GEPs of the genes in the TF cluster set (Table 2) and the TF binding pattern (TFBP) of the statistically best predictive (co)CRE for each gene based on their *p*-values (S3 Table). In case of ties between two or more predictors, the (co)CRE with the most regulatory input in terms of total number of bound TFs was considered. Gene-(co)CRE pairs with only one TF binding event were further removed. The method determines a clustering solution that minimizes an information criterion related to the standard Bayesian Information Criterion (*BIC* = *k*ln(*n*)−2ln(*L*)), where *k* is the number of parameters in the model, *n* the number of data points and *L* the maximized likelihood) or Akaike criterion (*AIC* = 2*k*−2ln(*L*)). In this case we used a criterion related to the latter, *AIC*2.5 = 2.5*k*−2ln(*L*), which was shown to be optimal in similar problems [64].

### Statistical significance of combinatorial binding of TFs

We calculated the statistical significance of multiple TF occupancy of *M* potential CREs as follows. Let the *i*^*th*^ TF (TF_*i*_) have *N*_*i*_ binding peaks overlapping the *M* CREs, then the probability that any CRE is occupied by at least one TF_*i*_ peak is ${1-(\frac{M-1}{M})}^{N_{i}}.$ The probability a CRE is occupied by at least one peak of each of *N* independently binding TFs is $\prod_{i=1}^{N}\left({1-(\frac{M-1}{M})}^{N_{i}}\right).$ The expected number of CREs occupied by at least one peak of each of the *N* TFs is $M\times \prod_{i=1}^{N}\left({1-(\frac{M-1}{M})}^{N_{i}}\right)$ and this was used with the Poisson distribution to obtain p values for the occurrence of CREs occupied by a combination of *N* TFs.

### Core regulatory network

TF gene set and TF cluster gene sets were used for generating two different types of network, namely the co-expression network (CEN) and cis-regulatory network (CRN). For the CEN, a pairwise Spearman correlation matrix (*M*) was generated for the gene expression values (GEP) in the set and an adjacency matrix (*A*) was derived from *M* where *A*_*ij*_ = 1 for the top 25% of the node pairs with highest correlation coefficient. The resultant matrix was represented by an undirected graph where an edge was drawn between two genes *i* and *j* if *A*_*ij*_ = 1. CRNs were generated with a directed edge from gene *i* to gene *j* if and only if they were connected in the CEN and gene *i* is a TF that binds to a chosen (co)CRE in the expression model for gene *j*. For generation of CRNs only selected CREs/coCREs with *q* ≤ 0.05 were used.

For computing the network parameters both CRNs and CENs are considered as undirected. A degree of a node is the total number of edges incident on it. For a node “*x*” in a network of *N* nodes, if “*o*” is the total number of all possible shortest paths, and if “*m*” is the total number of shortest paths from all *N*\*x* (all nodes except *x*) to *N*\*x* that traverse through *x*, then betweenness centrality is $\frac{m}{o}$. A node is termed as a neighbour of another node if there exists an edge between them and connectivity of a node is the total number of neighbours of the node. Neighbourhood connectivity of a node “*x*” is the sum of the connectivities of its neighbours [87, 88].

## Availability of data and materials

All the NGS based data are publicly available from Gene Expression Omnibus (GEO) with GSE69101 and GSE47950 accession numbers. These datasets are already published by Goode et al, 2016, Dev Cell (PMID: 26923725) and Wamstad et al, 2012, Cell (PMID: 22981692) respectively. The code is available in github as an R package (https://github.com/vjbaskar/lenhancer)

## Supporting information

S1 Table The three gene sets for which predictive models were generated and the gene expression data. (XLSX)

S2 Table Table containing CREs used in this study. (XLSX)

S3 Table Gene-wise predictive models for the genes and the chosen CREs/coCREs. (XLSX)

S4 Table Overlap between chosen CREs/coCREs and known enhancers. (XLSX)

S5 Table (A) Positive enhancer elements for the genes in Schutte et al. data and their overlap with predictions from our (co)CRE method and retrained JEME method. (B) Negative enhancer elements for the genes in Schutte et al. data and their overlap with predictions from our (co)CRE method and retrained JEME method.
(DOCX)

S1 Fig Gene expression analysis. (A) Principal components analysis of gene expression data where the cell types are projected on the first two principal components (PCs). (B) The cumulative contribution of the PCs to the variance observed. (C) Heatmap showing the hierarchically clustered cell types based on the correlation (Pearson) of their gene expression profiles. (D) BIC scores as a function of number of clusters (K) when clustering gene expression profiles for differentially expressed genes. The vertical line corresponds to the K with the lowest BIC score.
(EPS)

S2 Fig Gene sets used in this study. (A) The normalised expression values of the genes in the “*TF set*”. (B) shows the hierarchically clustered heatmap of the genes in the “*TF cluster set*”. (C) is a table containing the total number of genes that are differentially up (red) and down (blue) when comparing two cell types (rows to columns). See S1 Table for information on the genes used in this analysis.
(EPS)

S3 Fig Statistical overview of the cis-regulatory elements (CREs). Frequency of the CREs with a given number of chromatin events (A), size of CREs in (log_10_ of bp) (B), conservation scores (C), H3K27ac enrichment (D) and DNaseI-seq enrichment (E). (F) Gene specific models are built for the *TF Cluster* set with coCRE (post-coCRE) and without construction of coCREs (pre-coCRE, see Methods). A gene is considered if *p* < 0.05 in either of the two models. For a given gene the predictor with best drop in variance (*div*, from covariance test [49,50]) and *p* values (*pv*) for each of two models are considered and compared. The differences in the *div* and *p* values, Δ*div* and Δ*pv* respectively, were computed for each gene and the frequencies are plotted as bar charts (lower panel). The logged *div* and *pv* for both models for *TF Cluster* genes are plotted with lines coloured as given in the legend (upper panel). A +Δ*div* or a–Δ*pv* (red) indicates that the post-coCRE model is better than that of pre-coCRE and a–Δ*div* or a +Δ*pv* (blue) indicates vice versa. A paired t-test shows that post-coCRE models are significantly better than pre-coCRE models (*div*: 1.8e-06 and *pv*: 3.5e-06).
(EPS)

S4 Fig Gene expression models for the genes in the TF set. The gene-specific models showing the chosen coCREs and singleton CREs that are most predictive of the transcription factor’s gene expression profile. The method of generating the network and annotating the nodes and the edges is same as Fig 2A. S3 Table contains all the relevant details of the models and the CREs shown here. Please note that not all the TFs have supporting ChIP-seq data (Table 1). If a TF does not have ChIP-seq data in a given cell type, the value in that histogram is zero (see also Fig 2A)
(EPS)

S5 Fig Comparison of CREs/coCREs of key genes with known enhancer datasets. The genome browser snapshot for the tag density tracks and the datasets of known enhancers are generated similar to Fig 1D. The overlap between chosen (co)CREs (red bar below the density tracks) for Spi1 (A), Scl/Tal1 (B) and Erg(C) with Schütte et al. (blue and purple bars) are shown. (D) Browser snapshot showing enhancers predicted by JEME in the Hematopoietic Progenitors (HP) in blue, alongside the tested regions investigated by Schütte et al. in green and dark red, and CREs/coCREs selected by our method in red. (E) Overlap of predicted CREs of our method, and JEME, with Schütte et al (active).
(EPS)

S6 Fig Experimental testing of predicted enhancer for *Nfe2*. (A) Gene expression profile of *Nfe2* and chromatin accessibility profile of the predicted enhancer (inset). (B) Chosen CRE containing the predicted enhancer is highlighted as transparent cyan box. (C) Reporter gene investigation of the enhancer activity.
(EPS)

S7 Fig Network parameters for the GRNs. Network parameters such as the degree (A), Betweenness centrality (B) and Neighbourhood connectivity (C) for the key genes (*TF set*) (left) and all the genes (right) in the *TF Cluster set*. CRNs (yellow) and CENs (black) are generated as described in Methods section. (D) The co-expression networks (CEN) of genes in the *TF set*. The edges are drawn if the correlation coefficients (spearman) of GEP between two genes were in the top 25%. (E) Comparison of *p* values generated by the covariance test (covTest) and those from GEP randomisation for predictive models of the indicated TF genes.
(EPS)

S8 Fig Covariance tests’ significance values of CREs and coCREs in gene-wise models. The–log_2_P (adjusted) cutoffs on the x-axis and the total number of CREs or coCREs with the–log_2_P (adjusted) better than a given cut off for all the gene models with at least one β≠0 in blue and only for the significant models in red i.e. with q ≤ 0.05 (Table 2), on the y-axis.
(EPS)
