# Supplementary material for: Identification of gene specific cis-regulatory elements during differentiation of mouse embryonic stem cells: An integrative approach using high-throughput datasets
Source: PLoS Comput Biol. 2019 Nov 4;15(11):e1007337. doi: 10.1371/journal.pcbi.1007337 (PMC6855567; doi:10.1371/journal.pcbi.1007337)
Supplement: S5 Table — (A) Positive enhancer elements for the genes in Schutte et al. data and their overlap with predictions from our (co)CRE method and retrained JEME method. (B) Negative enhancer elements for the genes in Schutte et al. data and their overlap with predictions from our (co)CRE method and retrained JEME method. (DOCX) [file pcbi.1007337.s005.docx]

| **Gene** | **Enhancer** | **Predicted by JEME** | **Predicted by coCRE Method** | **HPC-7 TF binding** | **HP TF binding** |
| --- | --- | --- | --- | --- | --- |
| *Erg* | +65 | No | Yes | ERG, FLI1, TAL1 | CEBPβ,FLI1 |
| *Erg* | +75 | Yes | Yes | FLI1, PU.1 | None |
| *Erg* | +85 | No | Yes | FLI1, GATA2, GFI1B, RUNX1, TAL1 | FLI1, GATA2, GFI1, GFI1B, LMO2 |
| *Fli1* | -15 | Yes | No | FLI1, GATA2 ,GFI1B, RUNX1, TAL1 | FLI1,GATA2, GFI1 |
| *Fli1* | +12 | Yes | No | FLI1 | GATA1, PU.1 |
| *Gata2* | -93 | Yes | No | FLI1, RUNX1 | FLI1, LMO2, RUNX1 |
| *Gata2* | -92 | Yes | No | FLI1, PU.1 | PU.1 |
| *Gata2* | -3 | No | Yes | None | None |
| *Gata2* | +3 | No | Yes | FLI1, TAL1 | FLI1, GATA2, LMO2, TAL1 |
| *Gfi1b* | +13 | Yes | No | GATA2, GFI1B ,RUNX1, TAL1 | FLI1, GATA1, GATA2, GFI1, GFI1B, LMO2, TAL1 |
| *Gfi1b* | +16 | Yes | No | FLI1, GATA2, GFI1B, RUNX1, TAL1 | FLI1, GATA1, GATA2, GFI1, GFI1B, LMO2, RUNX1, TAL1 |
| *Gfi1b* | +17 | Yes | No | FLI1, GATA2, GFI1B, RUNX1, TAL1 | FLI1, GATA1, GATA2, GFI1, GFI1B, LMO2, PU.1, RUNX1, TAL1 |
| *Lyl1* | Promoter | Yes | Yes | FLI1, GATA2, GFI1B, PU.1, RUNX1, TAL1 | FLI1, GATA1, GATA2, GFI1, GFI1B, LMO2, PU.1, RUNX1, TAL1 |
| *Meis1* | +48 | Yes | Yes | FLI1, GATA2, GFI1B, PU.1, RUNX1, TAL1 | GATA1, GATA2, GFI1, GFI1B, LMO2, PU.1, RUNX1, TAL1 |
| *Spi1* | -14 | No | Yes | FLI1, GATA2, GFI1B, PU.1, RUNX1, TAL1 | CEBPβ, FLI1, GFI1, GFI1B, LMO2, PU.1, RUNX1 |
| *Runx1* | -59 | Yes | No | FLI1, GATA2, GFI1B, RUNX1, TAL1 | CEBPβ, FLI1, GATA1, GFI1, LMO2, TAL1 |
| *Runx1* | +3 | Yes | Yes | FLI1, GATA2, GFI1B, PU.1, RUNX1, TAL1 | FLI1, GATA1, GATA2, GFI1, GFI1B, LMO2, PU.1, RUNX1, TAL1 |
| *Runx1* | +23 | Yes | Yes | FLI1, GATA2, GFI1B, PU.1, RUNX1, TAL1 | CEBPβ, FLI1, GATA1, GATA2, GFI1, GFI1B, LMO2, PU.1, RUNX1, TAL1 |
| *Runx1* | +110 | Yes | Yes | FLI1, GATA2, GFI1B, RUNX1, TAL1 | CEBPβ,FLI1, GATA1, GFI1, GFI1B, LMO2, PU.1, TAL1 |
| *Runx1* | +204 | No | Not Considered | PU.1,RUNX1 | None |
| *Tal1* | -4 | No | Yes | None | FLI1 |
| *Tal1* | +19 | Yes | Yes | FLI1, GATA2, PU.1 | CEBPβ, FLI1, LMO2, PU.1 |
| *Tal1* | +40 | Yes | Yes | FLI1, GATA2, GFI1B, RUNX1, TAL1 | FLI1, GATA1, GATA2, GFI1, GFI1B, LMO2, PU.1, RUNX1, TAL1 |

**Table S5A. Positive enhancer elements for the genes in Schutte et al. data and their overlap with predictions from our (co)CRE method and retrained JEME method.**

**Table S5B. Negative enhancer elements for the genes in Schutte et al. data and their overlap with predictions from our (co)CRE method and retrained JEME method.**

| **Gene** | **Enhancer** | **Predicted by JEME** | **Predicted by coCRE Method** |
| --- | --- | --- | --- |
| *Erg* | +90 | No | No |
| *Erg* | +149 | Yes | No |
| *Fli1* | Promoter | No | Yes |
| *Fli1* | +2 | No | Yes |
| *Fli1* | +32 | No | No |
| *Gata2* | -83 | No | No |
| *Gata2* | -78 | No | No |
| *Gfi1b* | Promoter | Yes | No |
| *Lyl1* | -3 | No | Yes |
| *Lyl1* | +1 | Yes | Yes |
| *Meis1* | Promoter | No | No |
| *Meis1* | +69 | Yes | No |
| *Meis1* | +93 | Yes | No |
| *Spi1* | -18 | No | No |
| *Spi1* | Promoter | No | Yes |
| *Runx1* | -328 | No | Not Considered |
| *Runx1* | -43 | No | No |
| *Runx1* | -42 | No | No |
| *Runx1* | Promoter | No | Yes |
| *Runx1* | +24 | Yes | Yes |
| *Runx1* | +181 | No | Not Considered |
| *Tal1* | -9 | No | Yes |
| *Tal1* | Promoter | No | Yes |
| *Tal1* | +6 | No | Yes |
